# Patterns of Family Formation in Response to Sex Ratio Variation

**DOI:** 10.1371/journal.pone.0160320

**Published:** 2016-08-24

**Authors:** Ryan Schacht, Karen L. Kramer

**Affiliations:** Department of Anthropology, University of Utah, Salt Lake City, Utah, United States of America; University of Turku, FINLAND

## Abstract

The impact that unbalanced sex ratios have on health and societal outcomes is of mounting contemporary concern. However, it is increasingly unclear whether it is male- or female-biased sex ratios that are associated with family and social instability. From a socio-demographic perspective, male-biased sex ratios leave many men unable to find a mate, elevating competition among males, disrupting family formation and negatively affecting social stability. In contrast, from a mating-market perspective, males are expected to be less willing to marry and commit to a family when the sex ratio is female-biased and males are rare. Here we use U.S. data to evaluate predictions from these competing frameworks by testing the relationship between the adult sex ratio and measures of family formation. We find that when women are rare men are more likely to marry, be part of a family and be sexually committed to a single partner. Our results do not support claims that male-biased sex ratios lead to negative family outcomes due to a surplus of unmarried men. Rather, our results highlight the need to pay increased attention to female-biased sex ratios.

## Introduction

The concern that unbalanced sex ratios have a negative effect on family and social outcomes is well established in the literature [1,2]. Intellectual traditions, however, generate different predictions and draw different conclusions regarding the direction of the effect (reviewed in [3]). Of contemporary concern in both the popular and academic literature are the negative consequences of male-biased sex ratios. Commonly cited examples are drawn from India and China where, due to son preference and female-biased infanticide, there is a growing number of extra men (termed 'bare branches' [2]). Because men are more likely than women to be both victims and perpetrators of violence [4], and are typically characterized as the mate-seeking sex [5], their relative abundance at the population-level is expected to elevate conflict among males over partners, thus disrupting the formation of families, destabilizing pairbonds, and leaving many men unable to find a mate [2,6].

Given that sex-biased patterns of migration and mortality are currently altering demographic profiles in many regions of the world [7], the possible negative consequences of populations with too many men is of real, and not just academic concern. However, recent theoretical and empirical research challenges the expectation that male-biased sex ratios are associated with higher rates of male conflict, reproductive skew and family instability [8,9]. Central to this growing body of mating market research is that men’s willingness to marry and commit to a family is context-specific and responsive to pay-offs to varying reproductive strategies, in line with economic principles of supply and demand [1,8,10].

Here we evaluate family formation in the U.S. to determine whether male-biased populations are indeed associated increased instability. Our goals are first to investigate under which conditions men are more or less likely to marry and be part of a family and second to reconsider current concerns for social insecurity in populations with skewed sex ratios.

### Socio-demographic expectations of male-biased sex ratios

The influence that sex ratios have on relationship formation and mating systems has long been studied [11,12]. A slightly male-biased sex ratio at birth is characteristic of many nonhuman and human populations [13], yet sex ratios often become quite skewed in adulthood [14]. For example, large parts of China are projected to have a 15–20% excess of young men over the next several decades as a consequence of son preference and female-biased abortion and infanticide [15]. Because gender is one of the best individual-level correlates of violence [4], these statistics have raised considerable alarm. Of particular concern is the number of unmarried men who, mediated by elevated testosterone levels, tend to engage in more antisocial and violent behavior than married men [16]. And, the relative number of these unattached, risk-prone men is expected to grow with an increasingly male-biased sex ratio. Moreover, with partner rarity, males will face elevated levels of competition to secure a mate, leading to greater violent interactions between males, negatively impacting family outcomes [4] and societal stability [17]. This logic is also central to sexual selection theory. Increasing female rarity is theorized to produce greater male reproductive skew (i.e., a larger proportion of males are left unmated when females are rare) and favor more intense, antagonistic competition between abundant males over the limited number of females [18,19]. Thus, general expectations from both sociodemographic and traditional sexual selection theory are that a relative abundance of males will elevate levels of conflict (particularly between males over partners), reduce pairbond stability, and decrease paternal investment.

Studies evaluating these expectations, however, find that male-biased sex ratios are inconsistently associated with elevated rates of crime and violence [3,4,20]. Furthermore, an abundance of men has been found to be associated with higher rates of relationship commitment [9], monogamy [1,3,21], later age at first birth [22], less promiscuity in both sexes [23,24], and greater conjugal stability [25]. These results are consistent with recent work among nonhuman animals. For example, female rarity has been found associated with increased paternal investment [26] and decreased male promiscuity [27]. Therefore results from both human and nonhuman studies raise the question: does male excess at the population-level drive family instability or instead promote greater stability?

### Mating market expectations of male-biased sex ratios

A mating market approach reconsiders simple sex-based arguments (i.e., more men, more instability), and instead focuses on *variable* response to context [1]. Likewise, recent reformulations within sexual selection theory reconsider predictions drawn from sex differences in optimal mating rates and costs to reproduction [8], and instead emphasize that reproductive strategies are facultative in response to partner availability. The number of males and females in a population can be thought of as a mating market, which operates by supply and demand economics. The rarer sex has more bargaining power and can leverage their scarcity to realize their preferred mating strategy, while the more common sex caters to the preferences of the rarer sex in order to acquire a mate [1,10]. Thus, mating behavior is seen as a response to sex-structured payoffs to partner availability [8]. For example, mating market theory predicts that when males are rare they can behave more promiscuously, offer little parental investment and still be able to obtain partners. However, when women are in short supply, men will appeal more to female preferences and be more willing to commit to a single partner [1].

Here we use these frameworks to generate two sets of predictions with respect to family formation (Table 1). A socio-demographic approach expects lower rates of male marriage and family involvement at male-biased sex ratios. In contrast, a mating market approach expects the opposite. Using U.S. Census data, we analyze the relationship between the adult sex ratio (ASR; calculated as the number of men to women 15 to 45 years of age and over) and measures of family stability: marriage, nonmarital fertility, and female-headed households.

**Table 1:** Contrasting socio-demographic and mating market predictions of family formation outcomes in a population with a male-biased ratio. Specific measures are in parentheses.

	Socio-demographic	Mating market
**Male marriage:** (married %)	Lower	Higher
**Male family involvement:** (female headed house %)* (nonmarital fertility %)*	Lower	Higher

*lower % is associated with greater male family involvement & support for mating market predictions

## Methods

To test the competing theoretical predictions (see Table 1) we use county-level U.S. Census data [28]. U.S. data is ideally suited to evaluate our models for several reasons: 1) Counts are reliable and largely unhampered by many of the concerns of, for example, Chinese census data regarding biased reporting and unreliable measures [29]. 2) Predictor and response variables relating to marriage and family formation are publicly available. 3) Because most Americans either have married or plan to marry (only ~5% of adults are uninterested in ever marrying [30]), marriage counts are a reliable indicator of relationship preferences. 4) Women in the U.S. have autonomy when choosing their partners, and so family outcome measures are expected to vary with ASR values (bias in this association is likely present when using data from populations with a history of male patriarchal control of female reproductive options [31]).

### Study population

To explore family formation in response to ASR variation, we use U.S. census data disaggregated at the county level [28]. This level of data resolution is ideal for this analysis because it produces a large dataset (representing variation in sex ratios) where model covariates and outcome variables are available. Finer groupings of data aggregation (e.g., at the census tract level) result in data limitation and suppression. Higher groupings of data aggregation, while commonly used in the sex ratio literature (reviewed in [3]), have raised concerns about the ecological fallacy and drawing interferences of individual behavior from national or regional data [32]. Consequently, to avoid these concerns, we analyze data from counties and county equivalents (i.e., boroughs of Alaska and parishes of Louisiana) with available family outcome data and with ASR ranges from 55% female to 55% male (.8 to 1.2 respectively; ~90% of counties) for a total sample of 2,800 counties in 50 states.

The data source for our outcome variables is the 2010 U.S. Census American Community Survey (percent married, percent nonmarital fertility, and percent female-headed household [28]; Table 2). We define the ASR as the ratio of the number of males and females 15 to 45 years of age. We select this as the most appropriate measure of sex ratio for this analysis because it is inclusive of ages when males and females are most likely to marry and have children in the U.S.

**Table 2:** Descriptive statistics for outcome variables in the study sample of 2,800^*^ counties in 50 states.

*Variable*	*Mean (SD)*
**Men Married**	56.2% (7.0)
**Women Married**	53.0% (7.5)
**Nonmarital Fertility**	35.1% (18.4)
**Female Headed Household**	11.0% (4.3)

*data available from 2,782 counties for nonmarital fertility

### Statistical Approach

While we seek to keep our model set small and inclusion of covariates based in theory (following [33]), it is also important to account for possible within population sources of heterogeneity that may differentially affect family formation outcomes. To address these concerns, we fit multilevel models with state as a random effect and ASR, income (median household), and education (high school completion) as fixed effects. State is included to account for the nested structure of the data and likely clustering of county-level outcomes due to shared geography. Income and education are included because of the important role that socioeconomic status plays on family formation outcomes [34]. In sum, our fixed-effects measure stable contrasts between counties and the random effects allow for heterogeneity in the outcome measures by state.

Analyses were performed in R [35] and lme4 [36]. We employ multilevel models as the best analytic approach because 1) they are appropriate for nested data; 2) intercepts are allowed to vary by the group-level variable (random effect); and 3) fixed effects are shared across all groups. All statistical models include fixed effects for ASR, income, and education and a random effect for state and are constructed to assess the evidence for or against socio-demographic and mating market predictions (see Table 1).

## Results

**Fig 1:**
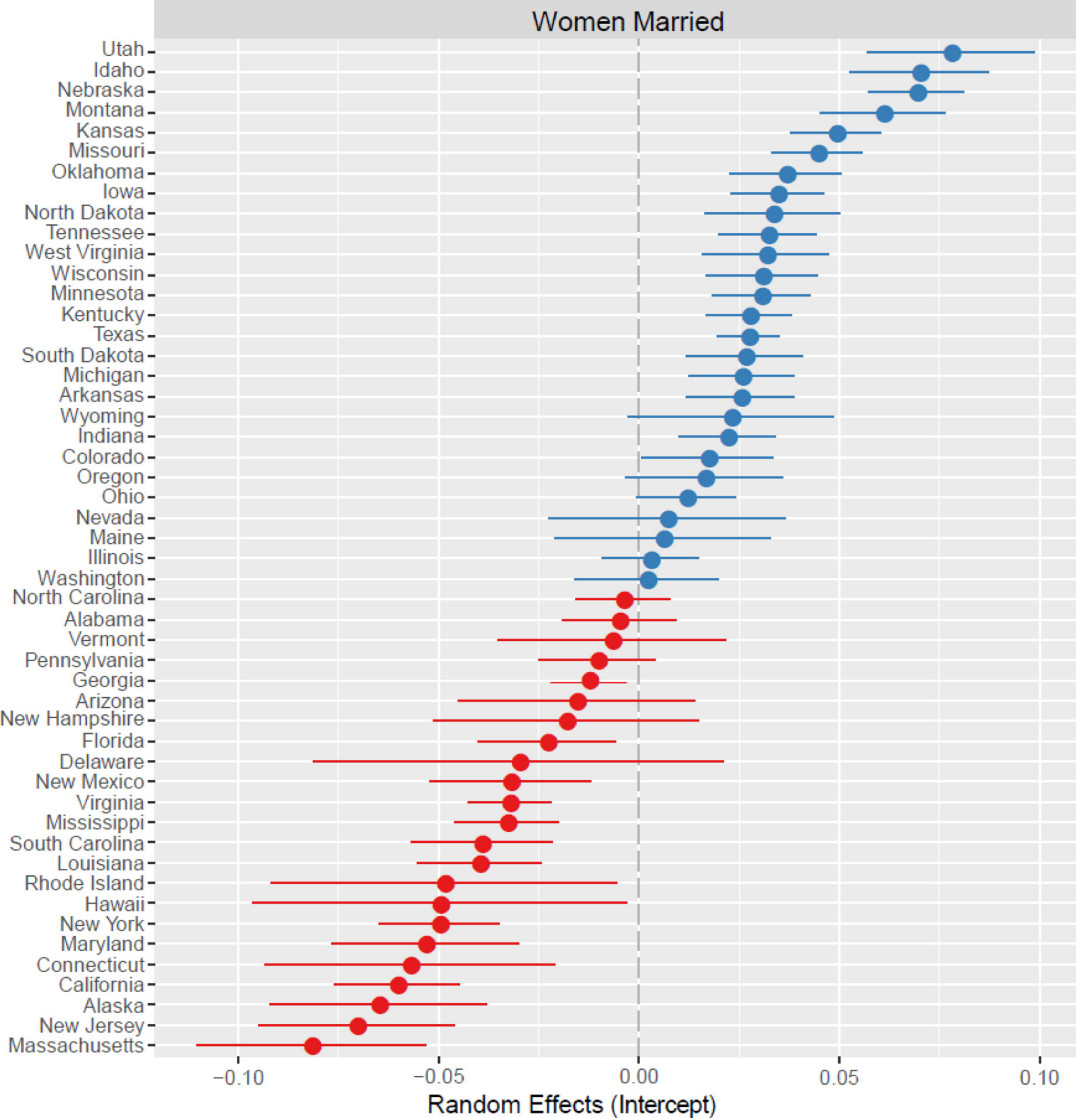
Plot of the random effects for the outcome variable Women Married. State-level deviations are shown relative to the mean (0.00; gray dashed line) with 95% confidence intervals around the intercept (closed circle). Blue circles represent intercepts higher than the mean and red circles lower than the mean.

Our analysis yields three main results. First, women are more likely to be married in counties where men are relatively abundant rather than rare (B = 0. 0031, SE = 0. 0002, p < 0.001; Table 3). Income also is positively associated with marriage, but we find no significant association with education. When evaluating the random effects, marital patterns vary by state (Fig 1). To explore clustering in our dataset across states, we calculate the intraclass correlation coefficient (ICC). The ICC is a measure of variance and describes how strongly those within a group resemble one another (0 not all, 1 identical [37]). The ICC is .34, indicating low to moderate clustering of counties by state.

**Table 3:** Model summaries and parameter estimates for the relationship between fixed and random effects and family formation outcomes.

	*Response*							
*Predictors*	Men Married		Women Married		Nonmarital Fertility		Female Headed Household	
	*Estimate*	*Std*. *Error*	*Estimate*	*Std*. *Error*	*Estimate*	*Std*. *Error*	*Estimate*	*Std*. *Error*
**Fixed Parts**								
(Intercept)	0.335989***	0.030317	0.089759**	0.029904	1.193086***	0.077098	0.479574***	0.015472
ASR	0.000851***	0.000208	0.003115***	0.000204	-0.003777***	0.000548	-0.001751***	0.000107
Income	0.000002***	0.000000	0.000002***	0.000000	-0.000004***	0.000000	-0.000000***	0.000000
Education	0.000458	0.000264	0.000024	0.000259	-0.003167***	0.000683	-0.001935***	0.000135
**Random Parts**								
σ2	0.0035		0.0034		0.0252		0.0009	
τ00, State	0.0014		0.0017		0.0021		0.0003	
ICCState	0.28		0.34		0.08		0.26	
R2	0.28		0.40		0.21		0.51	

Notes

* p < .05

** p < .01

*** p < .001

Models include county-level measures of *ASR*, *income* and *education* as fixed effects and *state* as a random effect. Estimates and standard errors are displayed for each response variable. The *intraclass correlation coefficient (ICC)* is a measure of variance and describes how strongly those within a group resemble one another (0: not at all, 1: identical). The terms σ^2^ and τ_00, State_ display the variance between counties and states respectively. Pseudo R^2^ values are also displayed.

**Fig 2:**
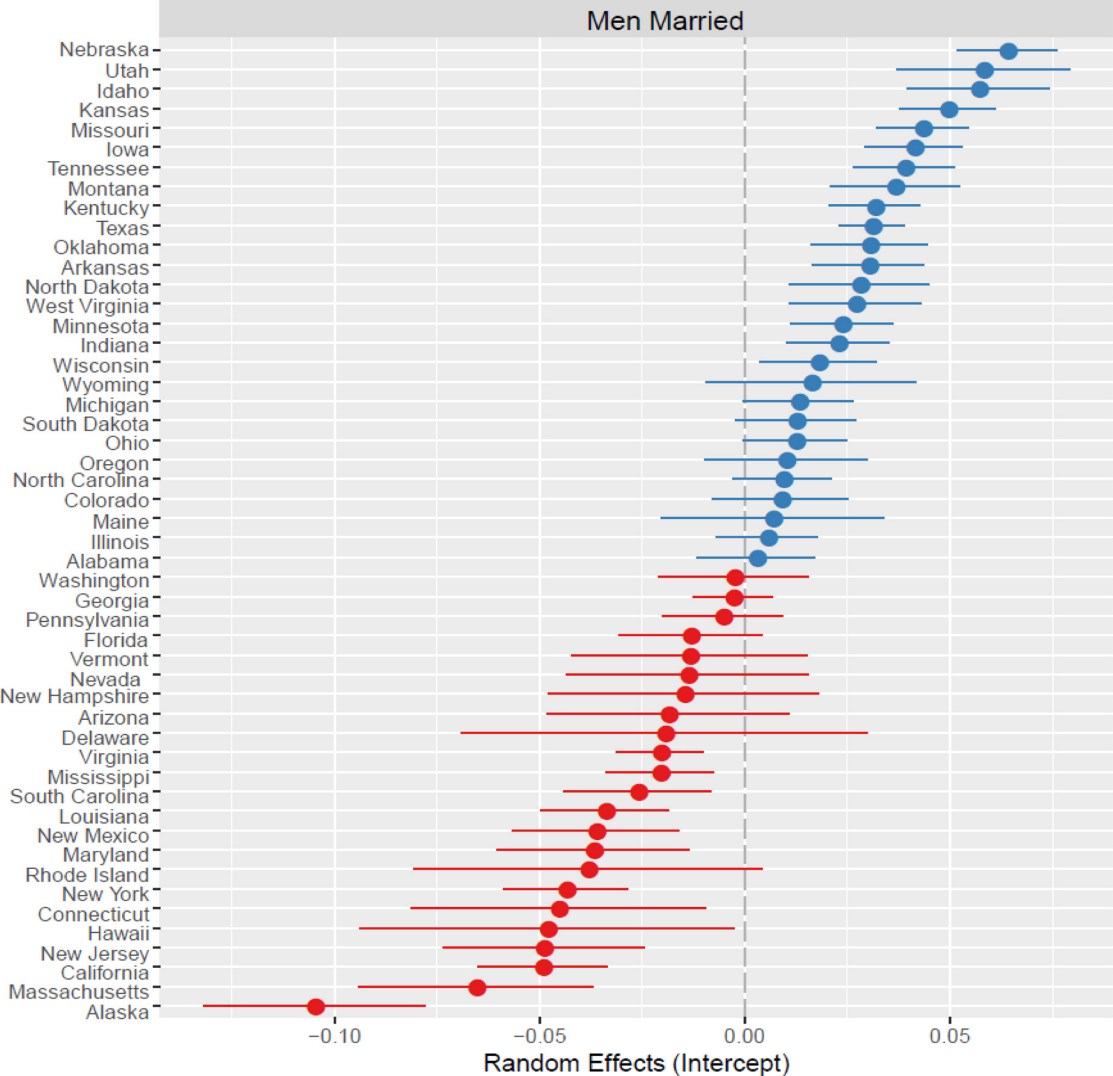
Plot of the random effects for the outcome variable Men Married. State-level deviations are shown relative to the mean (0.00; gray dashed line) with 95% confidence intervals around the intercept (closed circle). Blue circles represent intercepts higher than the mean and red circles lower than the mean.

Our second result is that the percent of married men likewise increases with male-biased ASRs (B = 0. 00085, SE = 0. 00021, p < 0.001). Men are *more* likely to be married when women are rare, rather than abundant. This suggests that when men are faced with partner abundance they alter their strategy and are less willing to enter into a committed relationship with a single partner. Thus, proportionately, unmarried men are more common at female-biased rather than male-biased sex ratios. With respect to the additional fixed effects, again income is positively associated with marriage, but education is unassociated, and marital outcomes vary at the state-level (Fig 2; ICC = .28).

**Fig 3:**
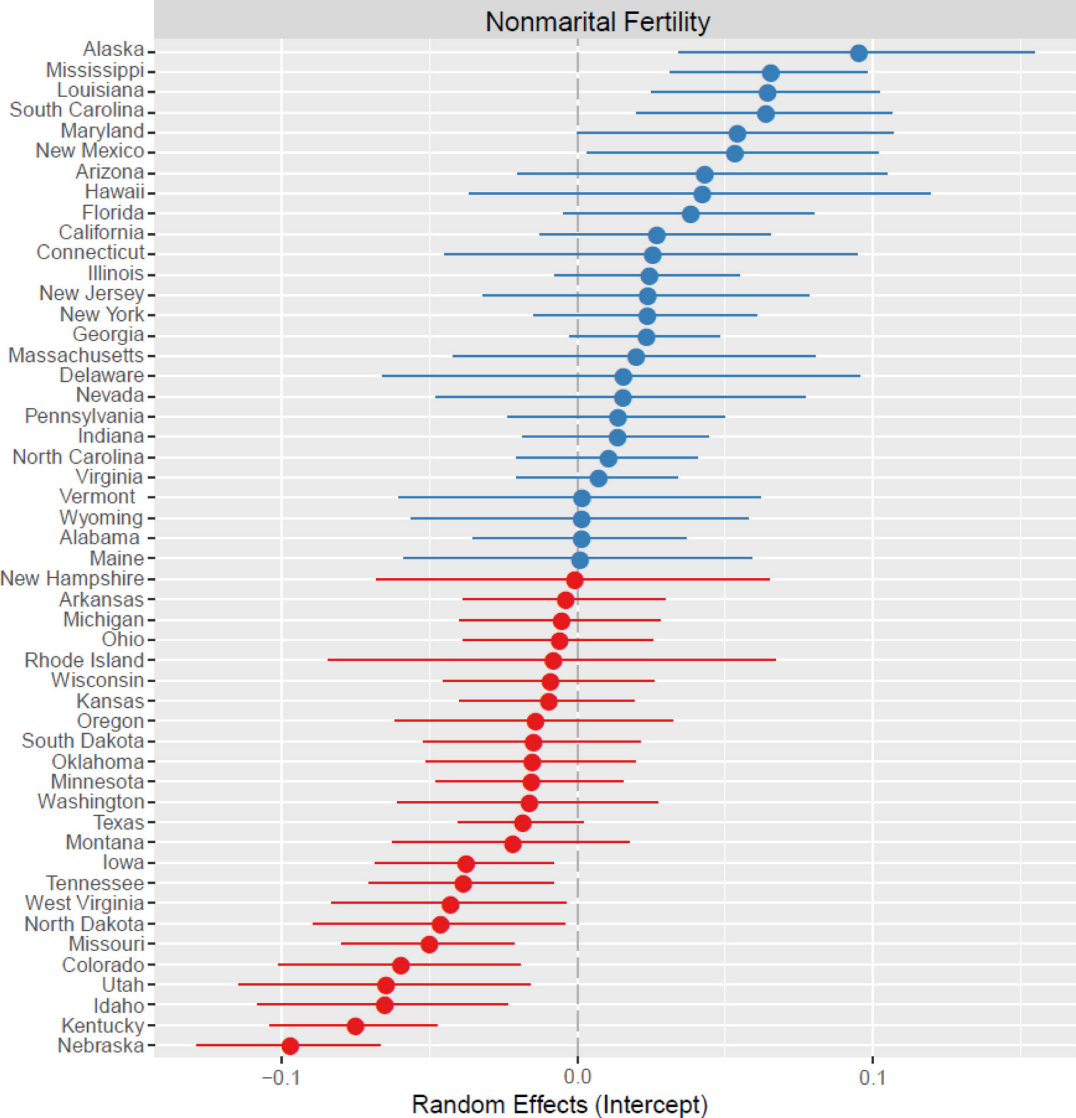
Plot of the random effects for the outcome variable Nonmarital Fertility. State-level deviations are shown relative to the mean (0.00; gray dashed line) with 95% confidence intervals around the intercept (closed circle). Blue circles represent intercepts higher than the mean and red circles lower than the mean.

**Fig 4:**
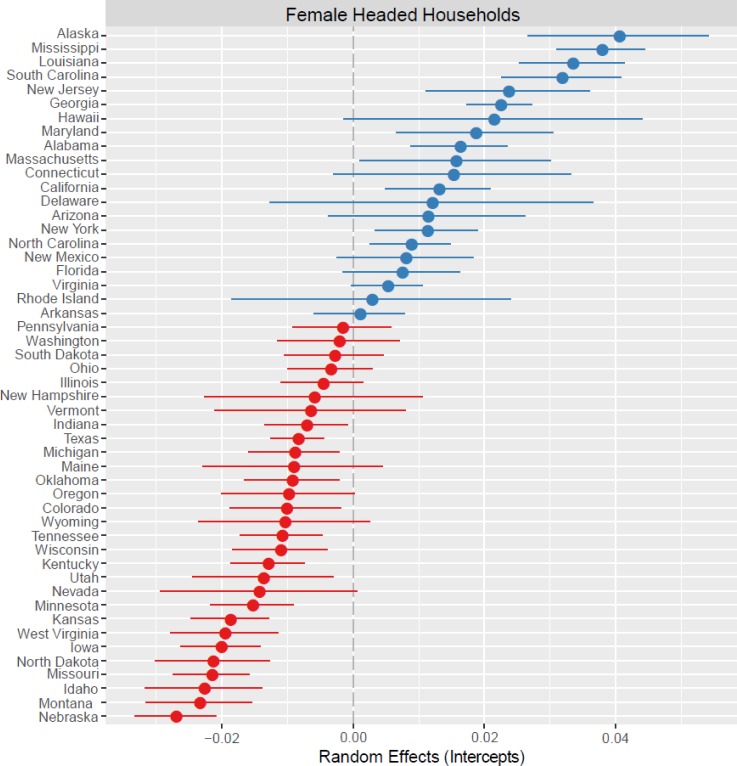
Plot of the random effects for the outcome variable Female Headed Households. State-level deviations are shown relative to the mean (0.00; gray dashed line) with 95% confidence intervals around the intercept (closed circle). Blue circles represent intercepts higher than the mean and red circles lower than the mean.

Third, using two frequently reported measures of family stability, we find that the frequency of both nonmarital fertility and female-headed households are highest in states with female-biased ASRs (B = -0.0038, SE = .0006, p < 0.01; B = -0.0018, SE = 0.0001, p < 0.001; Figs 3 and 4, respectively). Therefore, fewer children are born out of wedlock and households are less likely to be headed by single women in states with male-biased sex ratios. Income and education are both negatively and significantly associated with family stability measures. While the clustering of counties within states is evident for the outcome female-headed household (Fig 3; ICC = .26), it is much less important for the outcome nonmarital fertility (Fig 4; ICC = .08).

In sum, our analyses support two conclusions. First, the ASR is significantly associated with all family outcome measures, even after accounting for additional fixed and random effects. Second, for *all* measures of family formation, mating market predictions are supported. When sex ratios are male-biased, men and women are more likely to be married, fewer children are born out of wedlock, and fewer households are headed by women.

## Discussion

Both socio-demographic and mating market approaches emphasize the importance of sex-ratios in shaping family and societal outcomes [2,6,17,38]. Socio-demographic approaches predict that a male-biased sex ratio leaves many young men unable to find a mate and have a family. Mating market approaches, in general, predict the opposite. Men will be more willing to marry and have a family when partners are rare, and less willing to do so when they are abundant. Our results for all measures of family formation are consistent with mating market theory. Drawing from a multi-level dataset, we find that men are more likely to marry, be part of a family, and be sexually committed to a single partner when women are rare.

Below we discuss (i) the speculative nature of concerns that male-biased sex ratios generate family instability; (ii) support for our findings from nonhuman animal sex ratio research; (iii) implications for predicting male behavior, particularly that of unmarried men; (iv) applications of this study to future research.

Because the negative impacts of male-biased sex ratios, especially in Asian societies, tend to be based on anecdotal and historical accounts [2], the association between male abundance and family instability may be overstated [15]. Additionally, recent research in China suggests that the position of women has been elevated due to increased bargaining power in response to their relative rarity. Families are now having to provide more resources to enhance the attractiveness of sons [39] and divorce and remarriage rates for women are on the rise as women select more desirable partners [40]. These findings of positive outcomes for women in response to male abundance are not universal. However, they highlight the context-specific nature of mating strategies and the need to understand constraints to female autonomy by studying reproductive decision making comparatively. For example, male patriarchal control of women in parts of India seems to have intensified with a relative male abundance due to limitations on educational and economic opportunities for women [41]. Thus, while increasing evidence shows that marriages are more common and families are more stable in response to a shortage of women, the role women play in these decisions varies across populations.

Additionally, while female trafficking and prostitution in response to male-biased sex ratios are of public concern, supporting data are lacking. In China, areas with the least sex ratio bias have the highest proportions of sex workers [42]. Economic growth and socioeconomic inequality are better predictors of sex industry growth than a male-biased sex ratio. Moreover, STD rates are lowest in male-biased populations [43].

The socio-demographic approach emphasizes that the number of unmarried men will increase with female rarity. However, we find that percentages of men and women married are highest in U.S. counties with an excess of men. We interpret this to indicate that men are flexible in their desire to marry and are less *willing* to do so when they are rare and potential partners are abundant. These findings are in line with those from other studies across diverse animal taxa, including a recent analysis of 187 bird species [44], showing that male-biased sex ratios are associated with higher rates of pairbonding [45–49]. Consistent with mating market expectations [1] and frequency-dependent reformulations within sexual selection theory [8,50], males appear to leverage their rarity and pursue multiple partners when they are available. However, when potential partners are rare, males focus on acquiring and maintaining a single partner.

While an individual’s marital status is an important predictor of relationship strategy, not all single men are equally risk-prone and violently competitive. For example, recent work in China finds no evidence that unmarried men in male-biased areas are more violent. Instead unmarried men are more shy, withdrawn, and likely to be depressed [51]. This study suggests that unmarried males living in male-biased sex ratios are of much less concern when it comes to violence and other negative societal consequences than unmarried males living in female-biased sex ratios. When males are abundant and surrounded by competitors, rather than aggressively competing with each other, unmarried men may alternatively benefit from appealing to female preferences by investing in behaviors that signal their willingness to commit to marriage and parenting [52]. Indeed, recent research from China concludes that women have become increasingly demanding regarding a partner’s investment ability in response to an abundance of potential partners [39].

Although men may respond to female preferences when partners are rare, some concerns of ‘more males, more violence’ [2] are supported empirically [3]. Following mating market theory, we outline four predictions in which male-biased sex ratios would be associated with elevated levels of family and social instability.

First, understanding why a population is sex-biased and the conditions under which men and women seek relationships are key to predicting mating behavior. For example, many young men migrate to the U.S. state of Alaska for *short-term* labor opportunities. If temporary immigration self-selects for males who are not seeking committed relationships, they may pursue mating strategies that differ from males in other male-biased populations. Social stability may be very real in populations of itinerant males (e.g., areas of seasonal or temporary employment). Thus, we predict that male-biased populations, composed of short-term resident males, will be associated with higher rates of male aggression and violent conflict and lower rates of family stability than male-biased populations composed of long-term residents.

Second, threshold effects for mating strategies might exist at extremely unbalanced sex ratios. At very high male-biased sex ratios, males may shift from a strategy of courtship and appealing to female preferences, to one of antagonistic competition between males. Currently, this is the fear of what could occur in parts of China with a male surfeit [2,53]. While males might shift mating strategies at some sex ratio threshold, we are skeptical that this would occur under contemporary demographic conditions for three reasons. One, because sex ratios across China appear to have recently peaked and begun to decline, concerns for the future may be unfounded [14]. Two, recent work finds that male interest in long-term, committed relationships appears to increase with male-bias, even in populations approaching 150 men for every 100 women [9]. Three, nonhuman animal research finds that at sex ratios of nearly three males for every female, male-male conflict does not increase. Instead, males becomes increasingly willing to sacrifice themselves to be consumed by a female in exchange for a single mating opportunity [45]. Together, these points suggest either that males intensify their commitment to a strategy when abundant or that sex ratios need to be substantially more male-biased than they presently are to reach a threshold where males would pursue more aggressive behaviors.

Third, we predict that males with no chance of securing a partner may be particularly risk-prone and aggressive, regardless of the sex ratio. Such circumstances may arise when political or cultural systems exclude sectors of the male population from mating. In India, for example, the caste system and hypergyny may create a pool of males with no opportunities for marriage [54]. While regions with male-biased ASRs have the highest rates of homicide, these northern areas also rigorously enforce the caste system, complicating straightforward claims of association between sex ratio and violence in India [55,56]. In another example, Greenlaugh argues that governmental policies in China that suppress the marital options of rural bachelor males actually create the very pool of risk-prone, criminal males these policies are attempting to reduce as males must work outside of the law to secure a partner [57]. These examples suggest that behavioral polymorphisms in male mating strategies may also arise in response to *access* to the mating market, not just to partner availability.

Fourth, while our analysis indicates that males are more willing to be in committed relationships when partners are scarce, they may utilize violence to maintain a relationship. In the animal literature, the defense and control of a partner through male mate guarding can result in males directing violence at females [58]. We predict that intimate partner violence will be more common in populations where women are relatively rare because of more pronounced and frequent male mate guarding behaviors. Some evidence exists that rates of domestic abuse [59] and female homicide victimization by a partner [60] are higher at male-biased sex ratios. We emphasize that male aggression likely manifests itself in different ways across sex ratios because male mate acquisition strategies can take a variety of forms. Disaggregating measures of violence (intimate partner violence from sexual assault for example) may lead to a more productive understanding of the patterning of violent behavior [3].

Finally, we wish to point out that the social effects of *female-biased* sex ratios are generally under studied. Our analyses show that men are less likely to commit to a partner and family when women are abundant, not rare. It is under these circumstances that males may decrease investment in long-term relationships and focus on short-term mating strategies. When men are rare, rates of homicide and assault tend to be at their highest [61], possibly indicating that men are directing violence against other males over mating or partner opportunities [62]. Hormonal research also suggests that males tend to be more aggressive when they are around more women than men. A recent study found that when male U.S. college students compete in mixed-sex sport events, testosterone levels, which are linked with risk-taking and competitive behavior, vary significantly with team composition. When teams are female-biased men’s testosterone levels increase and when teams are male-biased they decrease, even after controlling for event outcome [63]. Together these studies suggest that productive insights and public policy recommendations could be gleaned from mating market theory. For example, consistent with the points above, ‘tough on crime’ policies in highly policed areas of the U.S. create extremely skewed adult sex ratios [23,24,64]. Our findings support previous research that attributes high rates of unstable families among poor and minority households to exogenous, rather than endogenous, factors. We show that family structure is sensitive to partner availability. Consequently, neighborhoods that are disproportionately affected by high incarceration rates and female-biased sex ratios are expected to express greater levels of family instability.

## Conclusions

Socio-demographic and mating market approaches to sex-ratio effects on family formation have important implications for public policy and popular media’s interpretations of unbalanced sex ratios. Predictions derived from the socio-demographic approach [2], while intuitive, are largely unsupported empirically. While it is true that men are more likely to be both victims and perpetrators of violence, they also behave variably in response to partner availability. Here we show that men are more likely to marry, be part of a family, and commit to a single partner when women are rare. Although male aggression might be elevated under certain circumstances (e.g., when men are excluded from the mating market or have short-term mating goals), it appears that it is female-biased rather than male-biased sex ratios that have negative effects on relationship and family instability.
